# Supplementary material for: Splice-Junction-Based Mapping of Alternative Isoforms in the Human Proteome
Source: Cell Rep. Author manuscript; Available in PMC 2020 Jan 15. (PMC6961840; doi:10.1016/j.celrep.2019.11.026)
Supplement: 2 [file NIHMS1546469-supplement-2.zip › DF1/PXD010154/Testis/Testis_56_SLC12A4_GTILAIITTSLVCILSQAWGR.pdf]

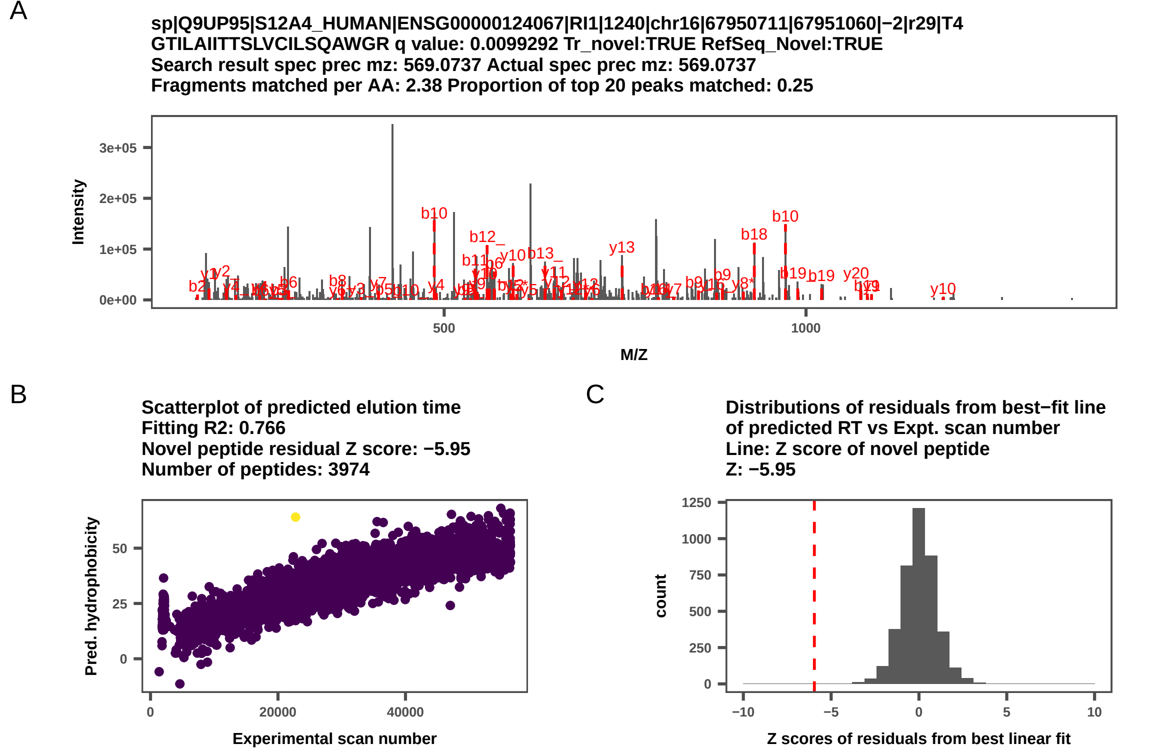
A
sp|Q9UP95|S12A4_HUMAN|ENSG00000124067|RI1|1240|chr16|67950711|67951060|−2|r29|T4
GTILAIITTSLVCILSQAWGR q value: 0.0099292 Tr_novel:TRUE RefSeq_Novel:TRUE
Search result spec prec mz: 569.0737 Actual spec prec mz: 569.0737
Fragments matched per AA: 2.38 Proportion of top 20 peaks matched: 0.25
Intensity
3e+05
2e+05
1e+05
0e+00
500
1000
M/Z
B
Scatterplot of predicted elution time
Fitting R2: 0.766
Novel peptide residual Z score: −5.95
Number of peptides: 3974
Pred. hydrophobicity
50
25
0
0
20000
40000
Experimental scan number
C
Distributions of residuals from best−fit line
of predicted RT vs Expt. scan number
Line: Z score of novel peptide
Z: −5.95
count
1250
1000
750
500
250
0
−10
−5
0
5
10
Z scores of residuals from best linear fit
